# Recurrent acute angle-closure attack due to plateau iris syndrome after cataract extraction with or without argon laser peripheral iridoplasty: a case report

**DOI:** 10.1186/s12886-016-0244-y

**Published:** 2016-05-26

**Authors:** Bonnie Nga Kwan Choy, Jonathan Cheuk Hung Chan, Carol Pui Yang Chien, Jimmy Shiu Ming Lai

**Affiliations:** Department of Ophthalmology, LKS Faculty of Medicine, The University of Hong Kong, Hong Kong SAR, Room 301, Block B, Cyberport 4, Hong Kong, People’s Republic of China; LKS Faculty of Medicine, The University of Hong Kong, Hong Kong SAR, Hong Kong, People’s Republic of China

**Keywords:** Plateau iris syndrome, Plateau iris configuration, Recurrent acute angle-closure attack, Argon laser peripheral iridoplasty, Cataract extraction

## Abstract

**Background:**

We describe two cases of recurrent acute angle-closure attack in patients with plateau iris syndrome after cataract extraction. Argon laser peripheral iridoplasty and cataract extraction have been used to reduce the occurrence of acute angle-closure attack in plateau iris syndrome although the risk cannot be completely eliminated. There is no consensus on the long term management of plateau iris syndrome. This is, as far as we know, the first case report of recurrent acute angle-closure attack in plateau iris syndrome after cataract extraction.

**Case presentation:**

We report two cases of recurrent acute angle-closure attack in 2 Chinese patients with plateau iris syndrome. The first patient was a 69 year-old woman who received bilateral argon laser peripheral iridoplasty and cataract extraction 2 years prior to the latest acute angle-closure with right eye intraocular pressure 48 mmHg. The attack was aborted medically. Peripheral iridotomy was patent and argon laser peripheral iridoplasty marks were mostly at peripheral 2/3 of the iris. Anterior segment optical coherence tomography confirmed bilateral plateau iris configuration. Use of long term pilocarpine or repeated argon laser peripheral iridoplasty to prevent recurrent angle-closure attack was discussed but she opted for observation. The second patient was a 64 year-old man presented with acute angle-closure after cataract extraction despite placement of laser peripheral iridotomy. Plateau iris syndrome was confirmed by anterior segment optical coherence tomography and he received argon laser peripheral iridoplasty.

**Conclusions:**

Acute angle-closure due to plateau iris syndrome can still occur despite previous cataract extraction and argon laser peripheral iridoplasty. These are the first reported cases of recurrent acute angle-closure attack due to plateau iris syndrome following cataract extraction, with or without previous argon laser peripheral iridoplasty. Repeated treatment with argon laser peripheral iridoplasty or pilocarpine could be considered although the long term efficacy is questionable. Argon laser peripheral iridoplasty should be applied as peripheral as possible so as to open up the drainage angle effectively.

## Background

Acute angle-closure (AAC) attack despite patent peripheral iridotomy (PI) is commonly due to plateau iris syndrome (PIS). This is frequently seen in subjects who have pre-existing narrow anterior chamber angles. Topical pilocarpine, argon laser peripheral iridoplasty (ALPI) and cataract extraction to open up the angles are options to reduce the occurrence of acute angle-closure attack in plateau iris syndrome [1]. However, the risk cannot be completely eliminated with such treatments and there is no consensus on the long term management of PIS.

We report two cases of recurrent AAC attack due to PIS in pseudophakic eyes. We would review the treatment strategies of PIS and discuss their pros and cons. This is, as far as we know, the first case report of recurrent acute angle-closure attack in plateau iris syndrome after cataract extraction.

## Case presentation

### Case 1

A 69 year-old Chinese woman first presented with left eye acute angle-closure with bilateral laser PI done in 1990. She subsequently developed another episode of bilateral acute angle-closure following pupil dilatation despite patent PI in January 2012 and was diagnosed as having PIS. Gonioscopy revealed bilateral appositional angle closure with no peripheral anterior synechiae, but no double hump sign was seen. Bilateral ALPI was performed and the attack was subsequently aborted. Since the angles were still narrow, bilateral cataract extraction was performed, right eye in February and left eye in April 2012. The intraocular pressure (IOP) remained in the range of teens in both eyes with no signs of glaucoma in subsequent follow up.

**Fig. 1:**
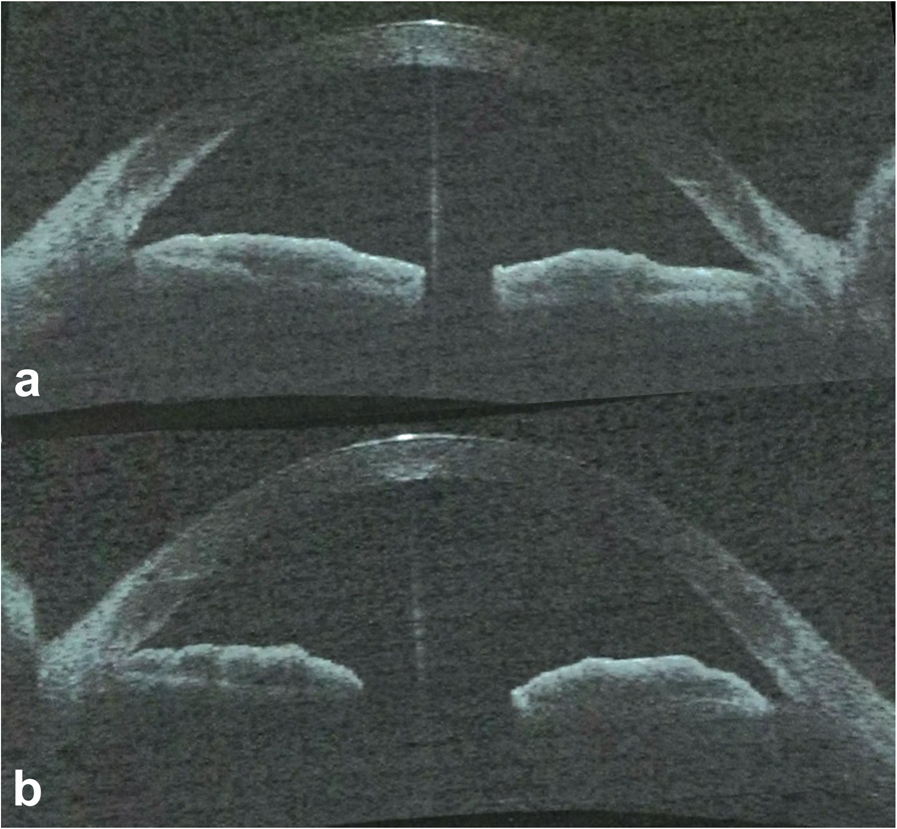
ASOCT images showing the angle configuration of right eye (the eye with acute angle-closure attack while on pilocarpine) (**a**) and the left eye (**b**) soon after the recurrent acute angle-closure attack

**Fig. 2:**
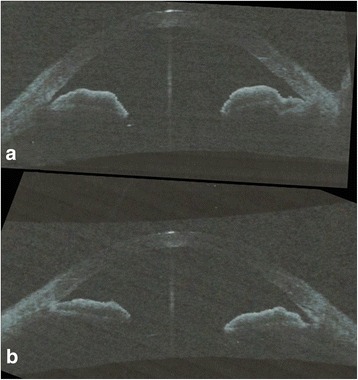
Repeated ASOCT images showing the angle configuration of right eye (**a**) and the left eye (**b**) after pilocarpine was stopped

In November 2013, she presented with right eye AAC (IOP 48 mmHg). The cornea was edematous, the anterior chamber was quiet and the central anterior chamber was deep. The PI was patent and the ALPI marks were mostly at peripheral 2/3 of the iris. Cup-disc ratio was normal. Gonioscopy was suboptimal due to poor corneal clarity but appeared to show grossly narrow angles with patchy closure. The attack was aborted medically. Anterior segment optical coherence tomography (ASOCT, SL-OCT (Heidelberg Engineering, Heidelberg, Germany)) confirmed bilateral plateau iris configuration (Figs. 1 and 2). Pilocarpine was initially prescribed to the right eye following the acute attack (Fig. 1) and was subsequently stopped (Fig. 2). We discussed with the patient concerning long term use of pilocarpine or repeat ALPI to prevent recurrent AAC attack. Since it was only her first attack after cataract extraction, she opted for observation.

### Case 2

A 64 year-old Chinese man with a history of bilateral diabetic retinopathy presented with left eye vitreous hemorrhage in January 2015. Left eye phacoemulsification with posterior chamber intraocular lens implantation, pars plana vitrectomy and endolaser was performed.

He subsequently presented with left eye AAC attack (IOP 44 mmHg) 3 weeks afterwards. Gonioscopy showed appositional angle closure with no synechiae formation nor neovascularization at the angles. Laser PI was subsequently performed. He developed another episode of AAC (IOP 64 mmHg) in March 2015 and was diagnosed as PIS by ASOCT. Left eye ALPI was performed and the attack was aborted. Post-ALPI ASOCT showed deepening of the angles and flattening of the iris. As right eye also showed plateau iris configuration on gonioscopy and ASOCT, right eye prophylactic ALPI was arranged to prevent occurrence of AAC.

## Discussion

We reported 2 cases of recurrent AAC in patients with PIS following cataract extraction in both cases, and after ALPI in the first case. The recurrence was postulated to be due to progressive anterior rotation of the ciliary body from its original anatomical position, especially in the first case where previous ALPI was not peripheral enough to open up the drainage angles. Despite a deepening of anterior chamber after cataract extraction, the drainage angles were still narrow, leading to IOP elevation.

PIS is not an uncommon cause of AAC attack. It is usually diagnosed after recurrent episode of acute IOP elevation following successful PI. It is further classified as complete (post-mydriatic IOP rise, as in our first patient in 2012) and incomplete syndrome (no IOP increase) [2]. PI is ineffective to open up the angles as the anatomy of the ciliary body and the angle remain unchanged in patients with plateau iris configuration [3], in contrast to cases of primary angle-closure due to pupillary block. AAC in PIS can be recurrent and not only does it pose symptoms of AAC to patients, it can damage the optic nerve, leading to glaucomatous optic neuropathy after recurrent attacks. Its diagnosis is aided by the gonioscopic finding of double hump configuration but it can be difficult to be seen, especially during acute attack due to presence of corneal edema. It can be more readily diagnosed with ultrasound biomicroscopy (UBM) and ASOCT which demonstrate the presence of an anteriorly inserted ciliary body and a flat iris configuration. The risk of recurrent attack may remain high in patients who demonstrate persistent narrow angle in ASOCT or UBM after ALPI, as in our first patient. Traditionally, UBM is used to assess anterior angle structures as in the case of PIS, which offers a high definition image and better penetration to deeper structures [4, 5]. ASOCT has been gaining popularity in recent years because of its advantages over UBM, including higher resolution, faster acquisition time, less operator-dependent, being non-contact with the ocular surface, and it does not require the subjects to lie down during examination [6]. Because of the advancement of ASOCT that it is capable of measuring other anterior segment parameters including corneal thickness and corneal curvature, it may become more readily available than UBM.

Treatment for PIS includes ALPI which aims to contract the peripheral iris root so as to pull open the anterior chamber angles. Previous study demonstrated that ALPI was effective in opening up the angles with long term follow up for at least 6 years [7]. However, it may fail with time as the angles become close again, especially if the laser is not applied close enough to the angle, such that the strength to mechanically open up the angles might be insufficient. Repeated ALPI can be an option although it is not certain whether patients failing ALPI would benefit from repeated ALPI. There was only one study suggesting that single repeated ALPI could effectively open up the angle following initial closure [7], but the study only involved 3 eyes whose angle closed again after one session of ALPI. There were no studies to demonstrate the efficacy of ALPI after cataract extraction.

Use of pilocarpine is another treatment option. It has been shown to decrease iris thickness and open up the angles in patients with PIS [8]. However, it is not without its side effects including headache (due to ciliary spasm), disturbed vision in dark and also it is reported to increase the risk of retinal detachment. Some patients may not be able to tolerate long term pilocarpine. Moreover, if it has be taken long term regularly by patients, it is going to have a negative impact on patients’ quality of life.

Cataract extraction can be considered in phakic patients. However, previous study showed that although cataract extraction could deepen the anterior chamber, it could not alter the angle structure [9]. Therefore, recurrent PIS can still occur following cataract extraction, as in our two patients. The acute elevation in IOP likely occurred as a result of impeded aqueous drainage at the angles due to the presence of anteriorly inserted ciliary body rather than due to pupil block or bulging lens which should have been eliminated by iridotomy and lens removal.

Other secondary cases of elevation in IOP should be sought for as recurrent PIS is not common after ALPI and cataract extraction. Causes include uveitis, Ponsner-Schlossman syndrome and peripheral anterior synechiae formation. In these cases, the use of pilocarpine and ALPI are not beneficial and they can potentially exacerbate the problem.

## Conclusions

We reported two cases of recurrent AAC in a patient with PIS following cataract extraction, one of them with previous ALPI. This is, as far as we know, the first case report of recurrent AAC attack after cataract extraction. As patients are still at risk of future AAC attack despite ALPI and cataract extraction, we should warn them about the symptoms of acute IOP rise. The patient should be monitored regularly to assess for progressive angle-closure. In patients with recurrent attacks, repeated treatment with ALPI or long term pilocarpine could be considered, although the long term efficacy of such interventions is unknown.

## Abbreviations

AAC, acute angle-closure; ALPI, argon laser peripheral iridoplasty; ASOCT, anterior segment optical coherence tomography; IOP, intraocular pressure; PI, peripheral iridotomy; PIS, plateau iris syndrome; UBM, ultrasound biomicroscopy
